# Exosomal microRNA signatures in multiple sclerosis reflect disease status

**DOI:** 10.1038/s41598-017-14301-3

**Published:** 2017-10-30

**Authors:** Saeideh Ebrahimkhani, Fatemeh Vafaee, Paul E. Young, Suzy S. J. Hur, Simon Hawke, Emma Devenney, Heidi Beadnall, Michael H. Barnett, Catherine M. Suter, Michael E. Buckland

**Affiliations:** 1 Department of Neuropathology, Royal Prince Alfred Hospital, Camperdown, NSW Australia; 2 Brain and Mind Centre, University of Sydney, Camperdown, NSW Australia; 3 Sydney Medical School, University of Sydney, Camperdown, NSW Australia; 4 School of Mathematics and Statistics, University of Sydney, Camperdown, NSW Australia; 5 Charles Perkins Centre, University of Sydney, Camperdown, NSW Australia; 6 Division of Molecular Structural and Computational Biology, Victor Chang Cardiac Research Institute, Darlinghurst, NSW Australia; 7 Faculty of Medicine, University of New South Wales, Kensington, NSW Australia; 8 Department of Neurology, Royal Prince Alfred Hospital, Camperdown, NSW Australia; 9 Present Address: School of Biotechnology and Biomolecular Sciences, University of New South Wales, Sydney, NSW Australia

## Abstract

Multiple Sclerosis (MS) is a chronic inflammatory demyelinating disease of the central nervous system (CNS). There is currently no single definitive test for MS. Circulating exosomes represent promising candidate biomarkers for a host of human diseases. Exosomes contain RNA, DNA, and proteins, can cross the blood-brain barrier, and are secreted from almost all cell types including cells of the CNS. We hypothesized that serum exosomal miRNAs could present a useful blood-based assay for MS disease detection and monitoring. Exosome-associated microRNAs in serum samples from MS patients (*n* = 25) and matched healthy controls (*n* = 11) were profiled using small RNA next generation sequencing. We identified differentially expressed exosomal miRNAs in both relapsing-remitting MS (RRMS) (miR-15b-5p, miR-451a, miR-30b-5p, miR-342-3p) and progressive MS patient sera (miR-127-3p, miR-370-3p, miR-409-3p, miR-432-5p) in relation to controls. Critically, we identified a group of nine miRNAs (miR-15b-5p, miR-23a-3p, miR-223-3p, miR-374a-5p, miR-30b-5p, miR-433-3p, miR-485-3p, miR-342-3p, miR-432-5p) that distinguished relapsing-remitting from progressive disease. Eight out of nine miRNAs were validated in an independent group (*n* = 11) of progressive MS cases. This is the first demonstration that microRNAs associated with circulating exosomes are informative biomarkers not only for the diagnosis of MS, but in predicting disease subtype with a high degree of accuracy.

## Introduction

Multiple sclerosis (MS) is the most common cause of neurologic disability in young adults^1^. MS is characterised by inflammation, demyelination, and neuro-axonal injury in the central nervous system, leading to progressive, long-term disability^1^. The clinical phenotypes of MS include relapsing-remitting MS (RRMS), and progressive forms: secondary progressive MS (SPMS) and primary progressive MS (PPMS)^2^. RRMS is the most prevalent MS subtype, comprising over 70% of cases. Within 10–15 years of disease onset, the majority of patients with RRMS will transition to SPMS, a phase of the disease defined by gradual clinical worsening that does not respond to any available treatment. PPMS is clinically indistinguishable from SPMS, except that it manifests *de novo*, without a preceding relapsing-remitting phase.

Currently there is no one definitive test for MS assessment; diagnosis and disease monitoring relies on multiple clinical parameters including clinical examination, magnetic resonance imaging, cerebrospinal fluid assessment, and electrophysiology^3^. Such investigations are not only costly over the protracted disease course, they also have limited utility in distinguishing active RRMS from progressive disease^2,4^.

Here we have assessed the utility of microRNAs (miRNA) within serum exosomes as biomarkers of MS disease. miRNA are small (18–25 nt) noncoding RNA with post-transcriptional gene regulatory function^5^. Exosomes are membrane bound vesicles shed by almost all cell types, and packed with small regulatory RNAs such as miRNA^6^. In many inflammatory diseases there is a significant increase in circulating exosome concentration^7,8^. Given that exosomes can cross the blood-brain barrier^9,10^, it is thus likely that at least some of the circulating exosomes in MS patients are derived from affected CNS cells or the associated inflammatory milieu.

We hypothesised that physiological changes associated with MS and its progression are reflected in differences in serum exosomal miRNAs. Using next-generation sequencing and integrative bioinformatics we found that serum exosome miRNA profiles can not only distinguish MS from healthy controls, but also distinguish RRMS from progressive forms of the disease with high accuracy.

## Results

### Serum exosomes carry a unique miRNA signature

**Figure 1:**
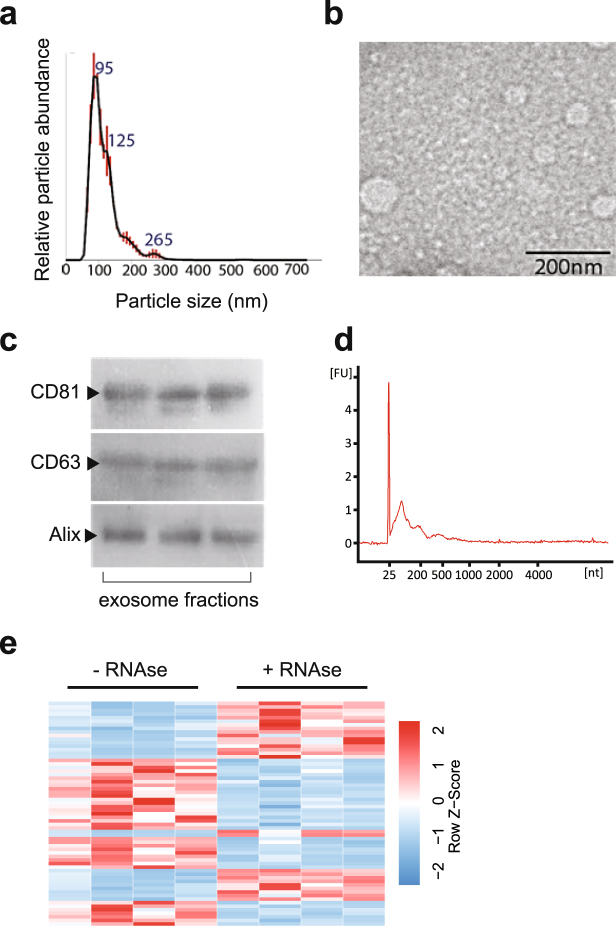
Identification and characterization of serum exosomes. (**a**) Size distribution of serum exosomes purified by size exclusion chromatography as analysed by Nanoparticle Tracking Analysis. (**b**) Transmission electron micrograph of serum exosomes demonstrates small vesicles with sizes ranging from 60–110 nm in diameter. (**c**) Western blotting for exosome-associated proteins CD63, CD81 and Alix in three separate patient samples (cropped images – uncropped originals available in Supplementary Figure 1). (**d**) Bioanalzyer trace of RNA extracted from serum exosomes reveals a predominant population of small RNAs without ribosomal RNA. (**e**) Hierarchical clustering of differentially expressed miRNAs shows that RNaseA treatment of serum results in unique miRNA population, (*p-*value ≤ 0.05 and fold change ≥ 2).

Patient blood was collected at the time of clinical consultation and pre-processed as detailed in the Methods. Exosomes were isolated from 1 ml of serum by size exclusion chromatography (SEC). Prior to exosome isolation, serum samples were treated with RNaseA to remove any unprotected circulating RNA. SEC fractions containing vesicles were pooled (fractions 8, 9, and 10; see Methods) and analysed by nanoparticle tracking analysis (Fig. 1a) and transmission electron microscopy (Fig. 1b). These analyses revealed a population of nanovesicles with a predominant size of 95 nm and cup-shaped morphology typical of exosomes. Western blotting of protein extracts for CD61, CD83 and Alix, confirmed that the particles isolated expressed all three characteristic exosome markers (Fig. 1c). RNA extraction from each sample yielded the typical RNA profile for exosomes, with the absence ribosomal RNA and enrichment of small (<200 nt) RNA species (Fig. 1d). Small RNA libraries were constructed from the exosomal RNA and sequenced to yield on average ~10 million reads per sample. Normalised miRNA read counts are detailed in Supplementary Data File 1.

To confirm that our protocols were selecting for small RNAs protected by association with exosomes, we compared miRNA profiles between four samples with and without RNAse pre-treatment. This identified 62 miRNAs whose relative expression differed significantly by at least 2-fold (Fig. 1e). This demonstrates that serum exosomes carry a distinct pool of protected miRNA that can be interrogated in MS diagnosis and progression.

### Exosomal miRNAs are dysregulated in MS patients and differentially expressed between disease subtypes

Twenty-five unrelated individuals with a diagnosis of MS (relapsing-remitting *n* = 14, progressive MS *n* = 11 (SPMS *n* = 7, PPMS *n* = 4)) and 11 healthy individuals were studied. A second, independent set of progressive cases (*n* = 11) was then analysed; participant demographic and clinical characteristics are outlined in Table 1. The healthy control cohort was selected to match for age and gender to the RRMS group. While progressive MS is associated with older age and different gender ratio compared to RRMS^11^, Pearson correlation demonstrates that age, gender and treatment did not correlate with the expression profiles of the identified miRNAs (Supplementary Figure 2a). Also, these clinical characteristics when incorporated to the multivariate modelling have minimal contributions to the model’s prediction accuracy (Supplementary Figure 2b).

**Table 1:** Characteristics of participants in this study.

Clinical Characteristics	RRMS (*n* = 14)	S/PPMS (Dis.) (*n* = 11)	HC (*n* = 11)	S/PPMS (Val.) (*n* = 11)
Age (mean ± SD)	42.5 (9.04)	53.4 (7.2)	40.3 (13.3)	52.7 (8.9)
Age of onset (±SD)	35.6 (7.28)	38.4 (8.5)	NA	32.3 (8.2)
Gender (F/M)	10/4	5/6	9/2	10/1
Disease Duration in years (±SD)	6.9 (7.1)	15 (9.4)	NA	20.4 (4.8)
Treatment (Y/N)	6/8	4/7	0/11	7/4
EDSS (±SD)	1.5 (1.0)	5.3 (1.6)	NA	6 (1.1)

Abbreviations: RRMS, Relapsing Remitting Multiple Sclerosis; S/PPMS, Secondary/Primary Progressive Multiple Sclerosis; HC, Health Control; Dis., Discovery set; Val., Validation set; EDSS, Expanded Disability Status Score; NA, Not Applicable.

We employed three statistical approaches (Student’s *t*-test, Fisher’s exact, Wilcoxon rank sum) to identify differential expression of miRNAs between healthy controls, RRMS, and progressive MS. miRNAs were identified as differentially expressed if they met a fold-change ≥ 2, and *p*-value ≤ 0.05 in at least two of the three statistical tests. Using this strategy we identified four significantly dysregulated miRNAs between healthy controls and RRMS patients, and a further four between healthy controls and MS patients with progressive disease (SPMS/PPMS; Table 2). These represent miRNAs that have the potential to be exploited as blood-based diagnostic markers.

**Table 2:** Significantly dysregulated miRNAs across all group comparisons.

	miRNA	CPM (B)	CPM (A)	FC	t-test	Exact test	Wilcoxon	Error rate
Control (A) vs. RRMS (B)	15b-5p	314	145.9	2.1	0.045	0.002	0.05	0.23
	30b-5p	673	246	2.7	0.06	0.0004	0.026	0.21
	342-3p	329	132	2.4	0.05	0.0002	0.008	0.21
	451a	39592	19114	2	0.009	0.0003	0.005	0.2
Control (A) vs. S/PPMS (B)	127-3p	1,715	752	2.2	0.007	0.001	0.003	0.17
	370-3p	707	321	2.2	0.008	0.002	0.007	0.18
	409-3p	2,893	1,385	2.1	0.005	0.002	0.002	0.17
	432-5p	682	308	2.2	0.002	0.001	0.003	0.16
	15b-5p	314	135	2.3	0.04	0.008	0.05	0.23
	223-3p	2646	934	2.8	0.026	0.002	0.047	0.22
	23-3p	1116	506	2.2	0.04	0.005	0.025	0.21
S/PPMS (A) vs. RRMS (B)	30b-5p	673	219	3.1	0.05	0.001	0.015	0.20
	342-3p	329	130	2.5	0.05	0.0016	0.02	0.22
	374a-5p	328	159	2.1	0.02	0.009	0.038	0.22
	432-5p	329	682	0.5	0.004	0.006	0.005	0.19
	433-3p	195	414	0.5	0.003	0.0027	0.0007	0.14
	485-3p	295	618	0.5	0.0056	0.002	0.004	0.17

Abbreviations: CPM, miRNA counts per million; FC, fold change; RRMS, Relapsing Remitting Multiple Sclerosis; S/PPMS, Secondary/Primary Progressive Multiple Sclerosis; HC, healthy control; EDSS, expanded disability status score; NA, not applicable; Error rate, estimated by leave-one-out cross validation.

We also compared miRNA profiles between the two clinically distinct MS subtypes, RRMS and progressive MS. Here we found nine miRNAs that were significantly differentially expressed between the two subtypes (Table 2). Importantly, *in silico* validation by leave-one-out cross validation correctly identified the test sample on average 80% of the time (range 77–86%; Table 2).

An independent validation set of 11 new progressive MS samples was then sequenced and analysed using the same methods. Differential expression analysis between this new group and healthy controls confirmed that three of the four original miRNAs (miR-370-3p, miR-409-3p, miR-432-5p) were significantly dysregulated. The fourth miRNA (miR-127-3p), while exhibiting close to 2-fold change in expression between the groups, failed to reach statistical significance (Table 3). Differential expression analysis between the validation group and RRMS samples identified eight out of nine significantly dysregulated miRNAs as identified previously (miR-15b-5p, miR-23a-3p, miR-223-3p, miR-374a-5p, miR-30b-5p, miR-433-3p, miR-485-3p, miR-342-3p, miR-432-5p) (Table 3).

**Table 3:** Significantly dysregulated miRNAs using progressive MS validation set.

	miRNA	CPM (B)	CPM (A)	FC	t-test	Exact test	Wilcoxon	Error rate
Control (A) vs. S/PPMS (B)	127-3p	402	752	0.53	0.08	0.03	0.07	0.25
	370-3p*	625	322	1.94	0.05	0.17	0.04	0.24
	409-3p*	2585	1385	1.87	0.002	0.0002	0.003	0.19
	432-5p*	589	309	1.91	0.03	0.6	0.03	0.23
	15b-5p*	314	110	2.8	0.017	7E-08	0.0004	0.17
	223-3p*	2647	675	3.9	0.011	0.0004	0.0005	0.15
	23a-3p*	1116	557	2	0.047	0.6	0.015	0.20
S/PPMS (A) vs. RRMS (B)	30b-5p*	673	90	7.5	0.014	2E-09	0.000001	0.00
	342-3p*	329	103	3.2	0.029	0.034	0.0007	0.17
	374a-5p*	328	188	1.7	0.033	6E-07	0.133	0.23
	432-5p*	329	589	0.5	0.051	0.0005	0.059	0.24
	433-3p*	195	492	0.4	0.006	1E-09	0.002	0.18
	485-3p	295	220	1.3	0.181	0.06	0.211	0.27

*miRNAs whose p-value < 0.05 in at least two tests and FC ≥ 1.7 in either directions. Abbreviations: *c*.*f*. Table 2.

### Serum exosomal miRNAs reflect MS subtypes

**Figure 2:**
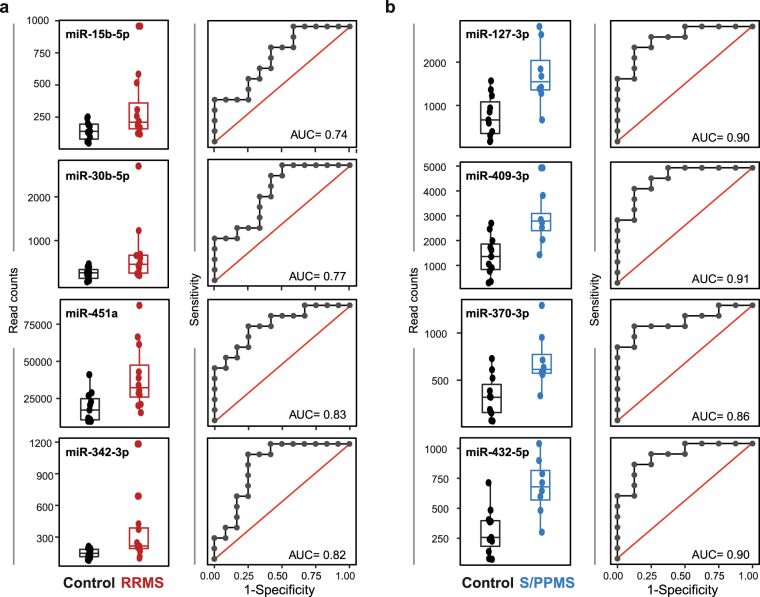
Differentially expressed miRNAs for control vs. RRMS or S/PPMS groups. Differentially expressed miRNA species were identified by Student’s t-test, Fisher’s exact test (EdgeR), and the Wilcoxon rank sum test for control versus RRMS (**a**) and control versus S/PPMS (**b**). MiRNAs with fold-change ≥ 2 and p-value ≤ 0.05 in at least two tests were identified as being differentially expressed. (left panels) Box-and-whisker plot for each miRNA species between the two groups (black box represents control group, red and blue boxes represent RRMS and S/PPMS respectively). (right panels) Logistic regression and receiver operator characteristic analysis performed on individual miRNAs to assess predictive power. Logistic regression was used to determine the linear model with the best discriminatory power between control and MS patient samples. The quality of this model was measured by the area under the curve (AUC) displayed on each plot.

**Figure 3:**
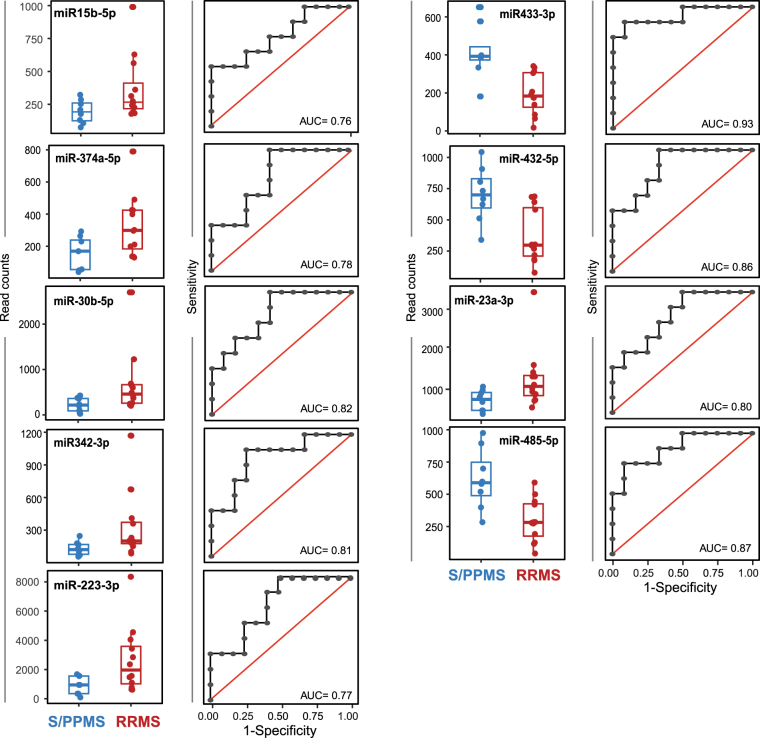
Differentially expressed miRNAs for RRMS vs. S/PPMS groups. Differentially expressed miRNA species were identified as per Fig. 2 above. (left panels) Box-and-whisker plot for each miRNA species between the two groups (red = RRMS group and blue represent S/PPMS group). (right panels) Logistic regression and receiver operator characteristic analysis of individual miRNAs to assess predictive power. Logistic regression was used to determine the linear model with the best discriminatory power between control and MS patient samples. The quality of this model was measured by the area under the curve (AUC) displayed on each plot.

We next examined the predictive power of each miRNA in our discovery sets using logistic regression (LR) models in which the predictor was the individual miRNA expression profile. Receiver operator characteristic (ROC) curves were determined for each candidate miRNA, where the true positive rate (sensitivity) is plotted against the false positive rate (1 – specificity). Area under the ROC curve (AUC) measures were ≥0.74 for each individual miRNA, for both RRMS and S/PPMS groups compared to healthy controls (Fig. 2); for RRMS compared to S/PPMS the AUC measurements were ≥0.76 (Fig. 3).

**Figure 4:**
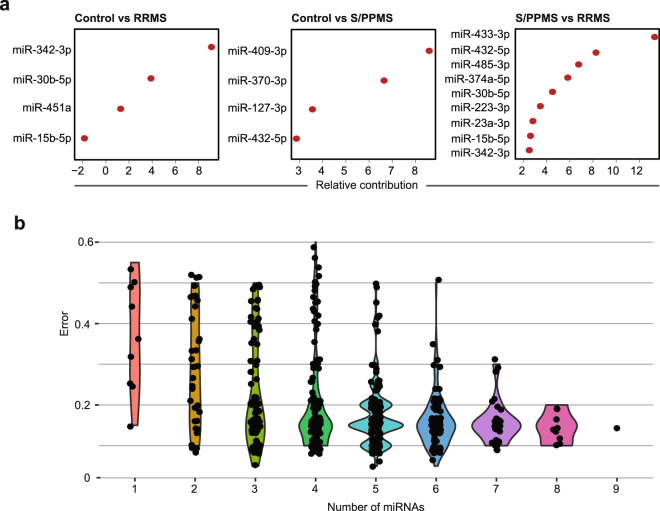
Random Forest multivariate analysis. (**a**) Significantly dysregulated miRNAs in each comparator group were ordered by the importance of contribution towards clinical classification as measured by Random Forest models. (**b**) Random Forest model was run using all possible combinations of dysregulated miRNAs to find combinations (i.e., signatures) with highest multivariate predictive power. Error rates of different combinations were stratified by the number of miRNAs (signature size) and their distributions were displayed as violin plots. This Figure shows results achieved in RRMS vs S/PPMS comparisons. Similar analyses were performed for other comparator groups and summarized in Table 4.

The relative importance of each miRNA in our discovery sets, when considered individually, was calculated using the Random Forest method and these shown in Fig. 4a. Multivariate analyses using Random Forest were used to determine whether the combined expression patterns of multiple miRNAs could improve this predictive power. All possible miRNA combinations in each comparator group were trialled; the corresponding Random Forest multivariate models were then generated and out-of-bag error rates estimated. Using these methods, we were able to achieve predictive power of 66% for RRMS and progressive MS versus controls. Strikingly however, a combination of 3 or more miRNAs provided a predictive power of 95% for distinguishing RRMS from progressive MS (Table 4 and Fig. 4b).

**Table 4:** miRNA combinations improve discriminatory power between relapsing-remitting (RRMS) and progressive (S/PPMS) disease.

# of miRNAs	miRNA composition	Error
9	miR-15b-5p, miR-23a-3p, miR-223-3p, miR-374a-5p, miR-30b-5p, miR-433-3p, miR-485-3p, miR-342-3p, miR-432-5p	0.15
6	miR-15b-5p, miR-23a-3p, miR-223-3p, miR-30b-5p, miR-485-3p, miR-432-5p	0.05
5	miR-23a-3p, miR-374a-5p, miR-30b-5p, miR-485-3p, miR-432-5p	0.05
5	miR-23a-3p, miR-223-3p, miR-374a-5p, miR-30b-5p, miR-485-3p	0.05
3	miR-223-3p, miR-485-3p, miR-30b-5p	0.05

We then examined the accuracy of Random Forest analysis in predicting the status of the validation set of new progressive samples using the same miRNA signatures. In this new test set, the original nine miRNAs reported for RRMS vs S/PPMS could predict 11/11 progressive MS samples in the validation sets (i.e., class specific error rate = 0%).

### Pathway analysis of dysregulated miRNAs

We performed functional analysis on targets of identified miRNAs. For each signature, we retrieved validated targets of miRNAs from three major miRNA-target datasets, miRecords^12^, miRTarBase^13^, and TarBase^14^ using the multiMiR R analysis package^15^. We then performed pathway overrepresentation analysis using KEGG pathways retrieved from the Molecular Signatures Database (MSigDB)-V 6.0^16^. Among top 5% of significantly enriched pathways (adjusted-p-value ≪10E-5), we observed relevant pathways such as neurotrophin signalling pathway, focal adhesion, and T cell receptor signalling. The top significantly enriched pathways are detailed in Supplementary Data File 2.

## Discussion

In this study we have used unbiased high-throughput sequencing on RNA derived from serum exosome preparations in order to capture the complete profile of these miRNAs in patient sera. We used size exclusion chromatography for exosome isolation; a method that is known for high purity of exosome extracts as well as high reproducibility^17^. This method, coupled with RNAse treatment of extracts, allows interrogation of exosomal-associated miRNAs; a source of biomarkers distinct from free circulating miRNA. Machine-learning approaches on miRNAs were used to examine their individual and collective predictive powers to identify disease subtype in MS. The results from this study confirm that exosome-associated miRNAs represent unique and potentially powerful biomarkers for this common neurological disease.

We have identified dysregulated miRNAs that discriminate healthy individuals from RRMS or S/PPMS patients with good predictive power. We also identified nine miRNAs that distinguish RRMS from S/PPMS patients with a very high degree of accuracy. A combination of just three miRNAs (miR-223-3p, miR-485-3p, miR-30b-5p) had a 95% accuracy rate of predicting disease progressive forms of MS from RRMS as identified by Random Forest analyses, suggesting that they may be useful clinical biomarkers. An independent validation set of progressive MS samples confirmed the reproducibility of our findings, and Random Forest analysis correctly categorised all samples in this new test set as progressive MS. To date, there are no clear clinical, imaging, or pathologic criteria to determine the point when RRMS converts to SPMS^2^. Our findings indicate that serum exosomal miRNA profiles may be a useful tool in assisting determination of this transition.

Some of the miRNAs we have identified have been previously implicated as circulating biomarkers in multiple sclerosis, namely miR-23a, miR-15b, miR-223, and miR-374a^4,18–25^. MiR-23a is involved in oligodendrocyte differentiation^26^ and increases within active and chronic MS lesions^23^. Also, both miR-23a and miR-15b target the fibroblast growth factor-2 (FGF-2) gene^27^. FGF2 is implicated in demyelination and remyelination, and there is some evidence that CSF FGF2 may be a useful marker of inflammation in MS^28^. MiR-223 is one of the few miRNAs that have been identified across several independent blood-based miRNA studies in MS^4^, and it targets the transcription factor STAT5 and other inflammatory regulators implicated in MS such as heat shock protein 90 and E2F^29–31^.

While several candidate miRNAs have been previously reported as potential MS biomarkers, the majority we have identified are novel. This likely reflects the unique constituent profile of exosomes versus free circulating miRNAs, and demonstrates that serum exosomal preparations represent a novel source of biomarkers. miR-451a was upregulated in RRMS patients compared to healthy controls; a miRNA previously reported as a regulator of oxidative stress with potential importance in a variety of neurodegenerative process^32^. We also identified miR-342-3p to be upregulated in RRMS patients; a miRNA especially enriched in microglia and dysregulated in Creutzfeldt-Jakob and Alzheimer’s disease^33–35^. Both miR-342-3p and mir-30b-5p have been proposed as free circulating miRNA biomarkers in Alzheimer’s and Parkinson’s diseases^27,28^, and their association with MS in this study suggests that they may be more general markers of neuro-axonal injury. Pathway analysis of transcripts known or predicted to be regulated by our candidate miRNA profiles yielded functional pathways highly relevant to MS disease pathogenesis such as neurotrophin signalling^36^, focal adhesion^37^, and T cell receptor signalling pathways^38^.

Small RNA analysis from biological fluids, including exosomal miRNAs, are subject to a variety of pre-analytical variables such as sample collection and processing methods, as well as differences in coagulation processes of serum and plasma^39,40^. This likely contributes to the only partially overlapping ‘free circulating’ miRNA profiles reported in different studies of MS to date^4,20^. We have used size exclusion chromatography for exosome isolation, and analyses of our extracts with nanoparticle tracking, western blotting and electron microscopy demonstrate that this isolation method yields highly enriched vesicle populations with characteristics of exosomes. In line with recommendations from The International Society for Extracellular Vesicles^41^, we have provided detailed technical information on our collection and isolation methodologies to allow comparison with future studies of serum exosomes in MS and other disorders. Our results with and without RNaseA treatment are in line with previous studies indicating that exosomes provide a protective environment for RNA^6^, and that some miRNAs appear to be selectively packaged in exosomes^42^.

In summary, this study demonstrates that exosomal-associated miRNAs have utility as biomarkers in MS. Our findings indicate that these biomarker profiles are distinct to those previously reported from serum or plasma circulating miRNA studies, while having comparable or superior predictive powers. Of note is the potential power to distinguish RRMS from progressive forms of the disease. The next generation of MS therapies offer the potential to specifically treat neuro-axonal and brain volume loss, and hence the ability to detect disease progression early may have major therapeutic and economic implications. If these exosomal biomarkers are able to indicate transformation to progressive disease earlier than current clinical methods, they are likely to have significant clinical utility. Longitudinal studies are needed to assess this question, and based on these initial investigations; these longitudinal studies should be pursued.

## Materials and Methods

### Participants

All patients attended the Royal Prince Alfred Hospital MS Clinic at the Brain and Mind Centre, University of Sydney. The study was ethically approved by the RPA Hospital Human Research Ethics Committee (#X13-0264), and all patients provided written informed consent. All methods were performed in accordance with the relevant guidelines and regulations. MS was diagnosed according to the revised McDonald criteria^43^, and SPMS patients were differentiated from the other clinical phenotypes (RRMS and PPMS) using the definitions offered by Lublin *et al*.^2^.

### Sample collection and preparation

A 20 mL blood sample was obtained from each participant’s using venepuncture with a 23 gauge butterfly needle. Blood was collected in three BD Vacutainer SST II Advance Serum-gel 7.5-ml Tubes (BD Vacutainer®, USA). Serum-gel tubes were left at room temperature for 30 minutes for coagulation, and then centrifuged at 1,800 g for 10 minutes. The resulting serum was transferred into 15 ml tubes and centrifuged at 3,000 g for 20 minutes to remove any cellular debris. The serum sample was aliquoted into 2 ml microcentrifuge tubes with O-rings (Interlab®, New Zealand), immediately snap-frozen in liquid nitrogen and stored at −80 °C. All serum-gel tubes were processed within 2 hours of collection.

### Exosome purification and characterisation

Serum (1 mL from each individual) was treated with RNase A at 37 °C for 10 min (100 ng/ ml, Qiagen, Australia) before exosome purification. The treated serum then underwent size exclusion chromatography (qEV iZONE Science) by being overlaid on qEV size exclusion columns followed by elution with 5 ml freshly filtered PBS. Ten fractions of 500 μl each were collected and analysed with Nanoparticle Tracking Analysis (NanoSight, Amesbury, UK). Fractions 8, 9, and 10 were pooled and stored at −80 °C for downstream analysis.

### Western Immunoblotting (WB)

Purified exosomes were resuspended with 4X sodium dodecyl sulfate (SDS) loading buffer and heated at 95 °C for 5 min to lyse. Samples were resolved on 12% (w/v) SDS-polyacrylamide gel electrophoresis (SDS-PAGE) and were transferred onto polyvinylidene difluoride (PVDF) membrane at 400 mA for 1 h using Criterion™ Blotter (BioRad, Hercules, CA, USA). Membranes were blocked in TBS-T containing 5% skim milk (w/v) followed by overnight incubation at 4 °C with a primary antibody (CD63, Abcam, ab193349, CD81, ProSci, 5195, Alix, Cell Signaling 21715). Membranes were washed with TBS-T (triplicate, 5 min) and incubated with a secondary antibody (conjugated to horse-radish peroxidase (HRP)) for 1 h at room temperature followed by three more TBS-T washing steps. Immunoreactive bands were visualized with enhanced chemiluminescence (ECL) (Amersham Biosciences, Inc.) detection reagent and imaged manually using X-ray film.

### Transmission electron microscopy

10 ul of purified exosomes were loaded onto carbon-coated, 200 mesh Cu formvar grids (#GSCU200C; ProSciTech Pty Ltd, QLD, Australia) and fixed with 2.5% glutaraldehyde in 0.1 M phosphate buffer (pH 7.4). Samples were negatively stained with 2% uranyl acetate for 2 min and dried overnight. Then samples were visualised at 40, 000 X magnification on a Philips CM10 Biofilter TEM (FEI Company, OR, USA) equipped with an AMT camera system (Advanced Microscopy Techniques, Corp., MA, USA) at an acceleration voltage of 80 kV.

### RNA extraction

Purified exosomes were processed for RNA extraction using the Plasma/Serum Circulating & Exosomal RNA Purification Mini Kit (Norgen Biotek, Cat. 51000) according to the manufacturers protocol. To check the yield, quality and size of extracted total RNA we analysed samples with an Agilent 2100 Bioanalyser (Agilent Technologies, United States) on a Eukaryote Total RNA chip.

### Small RNA sequencing

Sequencing libraries were constructed from exosome RNA using the NEBNext Multiplex Small RNA Library Prep Kit for Illumina (BioLabs, New England) according to the manufacturer’s instructions. Yield and size distribution of resultant libraries were validated using Agilent 2100 Bioanalyzer on a High-sensitivity DNA Assay (Agilent Technologies, United States). Libraries were then pooled with an equal proportion for multiplexed sequencing on Illumina HiSeq. 2000 System at the Ramaciotti Centre for Genomics.

### Data pre-processing and differential expression analysis

Data pre-processing was performed using a pipeline comprising of adapter trimming (cutadapt), followed by genome alignment to human genome hg 19 using Bowtie (18 bp seed, 1 error in seed, quality score sum of mismatches < 70). Where multiple best strata alignments existed, tags were randomly assigned to one of those coordinates. Tags were annotated against mirBase 20, and filtered for at most one base error within the tag. Counts for each miRNA were tabulated and adjusted to counts per million miRNAs passing the mismatch filter. Samples with low miRNA read counts (<50,000) and miRNAs with low abundance (<100 read counts across more than 50% of samples) were removed (two RRMS and three S/PPMS samples). Differential expression analysis was performed using three different statistical hypothesis tests including a non-parametric two-sample Wilcoxon test and two parametric tests – Student’s t-test, and an exact test (implemented in Bioconductor EdgeR) which tests for differences between the means of two groups of negative-binomially distributed counts. Data pre-processing and differential expression analysis were performed using Bioconductor and R statistical packages.

### Univariate analysis

We performed logistic regression (LR) and receiver operator characteristic (ROC) analysis to assess the predictive power of individual miRNAs between the two groups of interest. LR was used to identify linear predictive models with each miRNA as the univariate predictor. The quality of each model was depicted by the corresponding ROC curve, which plots the true positive rate (i.e., sensitivity) against the false-positive rate (i.e., 1-specificity). The area under the ROC curve (AUC) was then computed as a measure of how well each LR model can distinguish between two diagnostic groups. We then used leave-one-out cross-validation (LOO-CV) to estimate the prediction errors of the LR models. LOO-CV learns the model on all samples except one, and tests the learnt model on the left-out sample. The process is repeated for each sample and the error rate is the proportion of misclassified samples. Overall, cross validation is a powerful model validation technique for assessing how the results of a statistical analysis can be generalized to an independent dataset^44^. These analyses were performed using R stats (glm) and boot (cv.glm) packages.

### Multivariate analysis

The predictive power of multiple miRNAs as disease multivariate signatures was assessed using Random Forest (RF) modelling. RF modelling is an ensemble learning method for classification/regression that operate by constructing a multitude of decision trees at training time in order to correct for the overfitting problem^45^. We used the R RandomForest package which reports out-of-bag (OOB) error as an unbiased estimate of the test set prediction error. The model computes the ‘importance’ of each predictor by permuting OOB data that is for each tree the misclassification error rate on the out-of-bag portion of the data is recorded. The same procedure is done after permuting each predictor variable. The difference between the two are then averaged over all trees, and normalized by the standard deviation of the differences.

## Electronic supplementary material

Supplementary Information

Supplementary Dataset 1

Supplementary Dataset 2
