# Supplementary material for: Exosomal microRNA signatures in multiple sclerosis reflect disease status
Source: Sci Rep. 2017 Oct 30;7:14293. doi: 10.1038/s41598-017-14301-3 (PMC5662562; doi:10.1038/s41598-017-14301-3)
Supplement: Supplementary file 1 — Supplementary Information [file 41598_2017_14301_MOESM1_ESM.pdf]

SREP-17-07991A: "Exosomal microRNA signatures in multiple sclerosis reflect disease status"

Saeideh Ebrahimkhani, Fatemeh Vafaee, Paul Young, Suzy SJ Hur, Simon Hawke, Emma Devenney, Heidi Beadnall, Michael H Barnett, Catherine M Suter, Michael E Buckland

ID	Subtype	Gender	Age	Age of onset	Disease duration	Treatment	EDSS
**1**	HC	F	63	—	—	—	—
**2**	HC	F	55	—	—	—	—
**3**	HC	M	38	—	—	—	—
**4**	HC	F	53	—	—	—	—
**5**	HC	F	37	—	—	—	—
**6**	HC	F	19	—	—	—	—
**7**	HC	F	47	—	—	—	—
**8**	HC	F	22	—	—	—	—
**9**	HC	F	23	—	—	—	—
**10**	HC	F	48	—	—	—	—
**11**	HC	F	37	—	—	—	—
**12**	RRMS	F	37	36	1	No	2.5
**13**	RRMS	F	58	27	10	No	0
**14**	RRMS	F	47	48	10	No	0
**15**	RRMS	M	30	40	7	Dimethyl fumarate	1
**16**	RRMS	M	52	29	1	Fingolimod	2
**17**	RRMS	F	48	33	19	No	3
**18**	RRMS	F	34	26	22	Natalizumab	2.5
**19**	RRMS	M	46	30	4	Dimethyl fumarate	1
**20**	RRMS	M	30	43	3	No	2
**21**	RRMS	F	47	30	0	No	1.5
**22**	RRMS	F	45	40	7	No	3
**23**	RRMS	F	53	45	0	No	1
**24**	RRMS	F	27	36	17	Dimethyl fumarate	0
**25**	RRMS	F	46	26	1	No	2
**26**	SPMS (Dis.)	M	44	32	12	No	4
**27**	SPMS (Dis.)	M	47	44	3	Dimethyl fumarate	7.5
**28**	SPMS (Dis.)	M	52	19	33	Dimethyl fumarate	6.5
**29**	SPMS (Dis.)	M	67	37	30	No	3.5
**30**	SPMS (Dis.)	F	49	38	11	No	2.5
**31**	SPMS (Dis.)	F	52	30	22	No	3.5
**32**	SPMS (Dis.)	M	47	44	3	Fingolimod	6
**33**	PPMS (Dis.)	F	61	49	12	No	6.5
**34**	PPMS (Dis.)	M	60	42	18	Interferon beta-1a (Rebif)	6
**35**	PPMS (Dis.)	F	48	39	9	No	6
**36**	PPMS (Dis.)	F	61	49	12	No	6.5
**37**	SPMS (Val.)	M	37	22	15	Natalizumab	4.0
**38**	SPMS (Val.)	F	52	38	14	Natalizumab	6.0
**39**	SPMS (Val.)	F	46	23	23	Fingolimod	6.0
**40**	SPMS (Val.)	F	59	39	20	No	6.5
**41**	PPMS (Val.)	F	60	40	20	Dimethyl fumarate	7.5
**42**	SPMS (Val.)	F	37	17	20	Interferon beta-	6.5

						1b	
**43**	SPMS (Val.)	F	53	31	22	Peginterferon beta-1a	3.5
**44**	SPMS (Val.)	F	66	41	25	No	6.0
**45**	SPMS (Val.)	F	53	41	12	Glatiramer acetate	4.0
**46**	SPMS (Val.)	F	56	29	27	No	7.5
**47**	PPMS (Val.)	F	61	47	14	No	6.5

Supplementary Table 1: Detailed patient characteristics

Supplementary Figure 1: Uncropped Western bots for Alix, CD63, CD81

Alix:

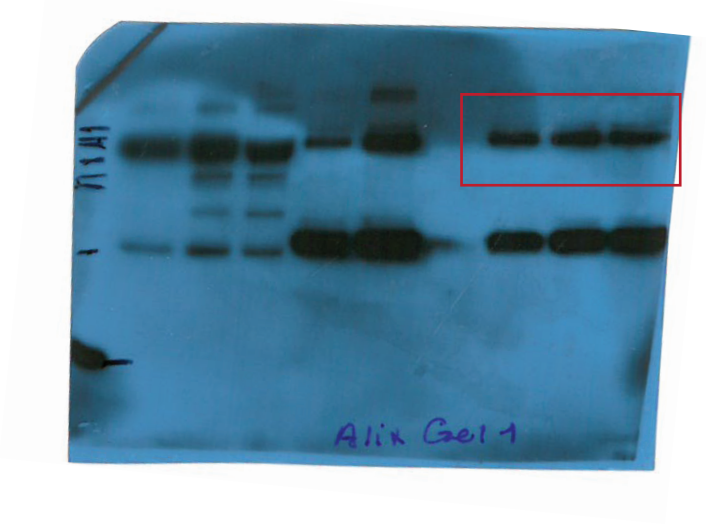

CD63:

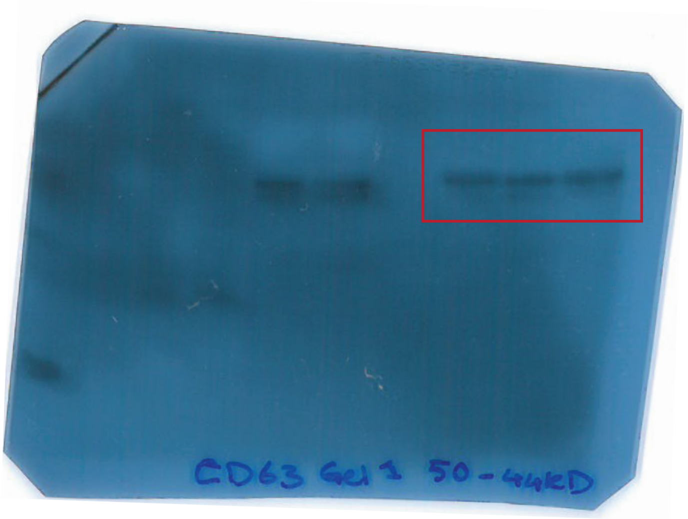

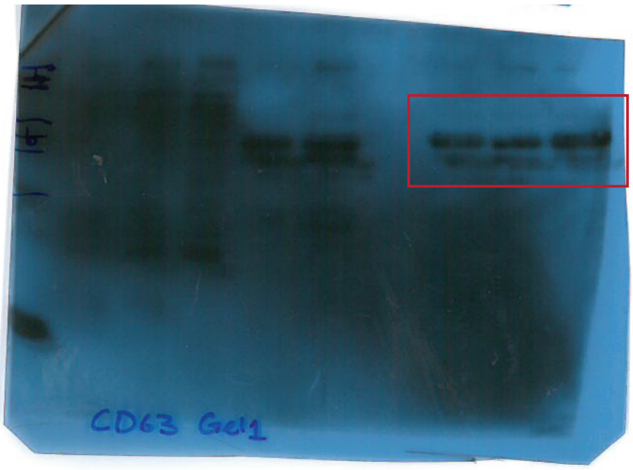

CD81:

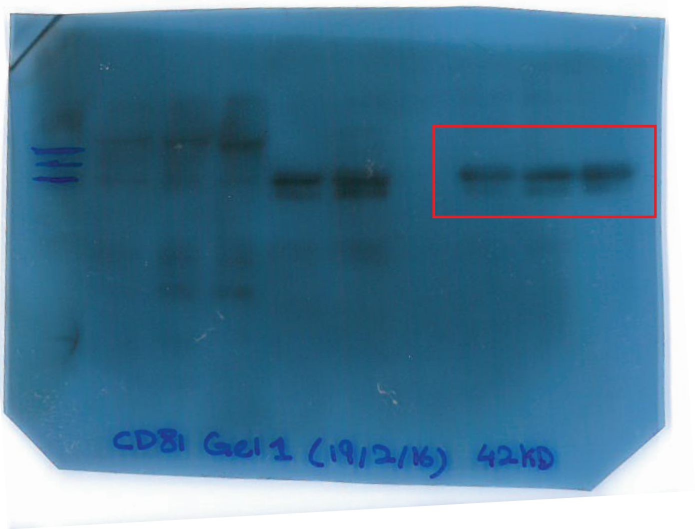

A: Correlation of individual miRNA with demographic variables

	age	gender	treatment
miR-23a-3p	0.099	0.347	0.017
miR-374a-5p	0.013	0.295	0.000
miR-433-3p	0.411	0.225	0.330
miR-223-3p	0.124	0.347	0.000
miR-15b-5p	0.095	0.347	0.052
miR-485-3p	0.559	0.295	0.277
miR-432-5p	0.384	0.277	0.382
miR-30b-5p	0.096	0.416	0.000
miR-342-3p	0.126	0.468	0.069
Average	***0.212***	***0.322***	***0.168***

B: Relative contribution of each variable

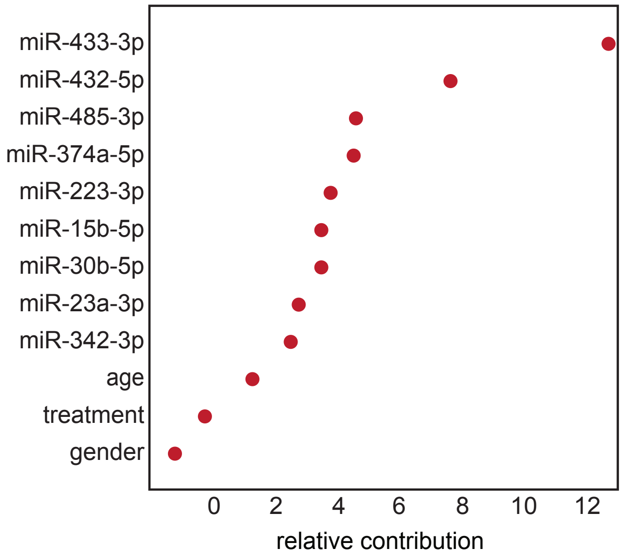

Supplementary Figure 2. A: Pearson correlation of age, gender and treatment with individual miRNAs show that these clinical variables are not correlated with the expression profiles of identified miRNAs across RRMS and S/PPMS samples (similar results attained using ranked-based correlation techniques, i.e., Spearman or Kendall).

B: age, gender and treatment were incorporated as predictors into the Random Forest nonlinear and multivariate model. The importance plot demonstrates the minimal contribution of these variables in predicting MS subtypes and illustrates the predictive power of signature miRNAs independent of these clinical characteristics.
